# Motivation for recreational sport participation and leisure constraints: a segmentation perspective

**DOI:** 10.3389/fspor.2025.1697603

**Published:** 2026-01-13

**Authors:** Apostolia Ntovoli, Georgia Stavropoulou, Thomas Karagiorgos, Yannis Lianopoulos, Garyfallos Anagnostou, Kostas Alexandris

**Affiliations:** 1 Department of Life and Health Sciences, School of Health Sciences, Limassol, Cyprus; 2 Laboratory of Management of Sports, Recreation, and Tourism, School of Physical Education and Sport Science, Aristotle University of Thessaloniki, Thessaloniki, Greece; 3 Sport Entrepreneurship and Innovation Lab, Department: School of Physical Education & Sport Science (Serres), Aristotle University of Thessaloniki, Thessaloniki, Greece

**Keywords:** barriers, latent class analysis, leisure constraints, motivation, recreational sport participation, recreational sports

## Abstract

**Introduction:**

Physical inactivity is a global problem today, with multiple negative impacts on physical and psychological health. This study used the self-determination perspective and the hierarchical model of leisure constraints to study recreational sport participation within the framework of the leisure negotiation proposition.

**Methods:**

Latent class analysis (LCA) was employed in order to cluster individuals according to their motivation level (intrinsic, extrinsic, and amotivation) and to compare the cluster groups' scores on intrapersonal, interpersonal, and structural constraints. The sample of the study included three hundred and eighteen (*N* = 318) individuals of the general population in Greece.

**Results:**

Three distinct segments emerged from the latent class analysis, based on the motivation scores. The study revealed that the cluster with the highest intrinsic and extrinsic motivation scores had the lowest intrapersonal constraints scores, providing support for the important roles of intrinsic and extrinsic motivation in successful negotiation and overcoming intrapersonal constraints.

**Conclusion:**

The role of extrinsic motivation should be noted, as it is equally important in the context of recreational sports, alongside intrinsic motivation, through the individual's internalization processes.

## 1 Introduction

Physical inactivity has emerged as a significant global public health concern. According to the World Health Organization (1), nearly one-third of adults globally (approximately 1.8 billion) remained physically inactive in 2022, marking a significant rise since 2010 and underscoring that physical inactivity has emerged as a major global public health concern. Notably, the prevalence of insufficient physical activity is markedly higher in high-income countries, where 36.8% of adults are inactive, compared to just 16.2% in low-income nations (2). At the European level, the most recent data from Eurostat (3) reveals worrying trends regarding physical activity among European Union (EU) citizens. Specifically, 45% of the EU citizens reported that they never engage in physical activities, whereas 38% exercise or play sports at least once per week. A smaller proportion—just 6%—reported engaging in physical activity five times a week or more. Rates of weekly participation in exercise or sport are highest in Finland (71%), Luxembourg (63%), the Netherlands (60%), Denmark (59%), and Sweden (59%). On the other hand, physical inactivity was reported to be most pronounced in countries such as Portugal (73%), Greece (68%), and Poland (65%), where over half of the respondents indicated that they never engage in any form of physical activity (56). Noting that Greece ranks second among EU member states in terms of physical inactivity levels underscores the critical need to promote active recreation and to design strategies that encourage physical activity among the general population (4–6). It should be noted that these statistics might have been influenced by the COVID-19 pandemic. Most published studies have shown that the Covid-19 pandemic negatively influenced participation in physical activity; however, there are also studies that showed positive influences (7). With the data that will be provided by the next Special Eurobarometer on sports, we will be able to draw some conclusions.

Several theoretical approaches and models have been developed to study physical activity participation. The current study used the Self-determination perspective (8, 9), the Leisure Constraints theory (10), and the subsequent Negotiation and Balance propositions (11) to study how constraints and motives interact and influence individuals' decisions to take part in recreational sport activities. The Negotiation proposition was developed as an advancement of the hierarchical model of leisure constraints (12). It proposes that, while all individuals face constraints, some of them are capable of successfully overcoming them and engaging in leisure participation, even in a modified form (e.g., a different type of activity or less frequently). The successful negotiation of this interaction was proposed to be determined by several factors, such as motivation (13), personality (14), perceived well-being (4, 5), and social support (15).

Using the negotiation proposition in this study, we aimed to examine the interaction between motivation and constraints to explain the negotiation process. While there has been some evidence that motivation and constraints relate (13), this relationship has not been examined at a detailed level, measuring motivation and constraints with multidimensional models. The Self-determination motivation theory (16) was employed in the present study, incorporating intrinsic, extrinsic motivation, and amotivation dimensions. Leisure constraints were also examined at a detailed level, measuring all three dimensions of leisure constraints, as proposed in the literature: intrapersonal, interpersonal, and structural.

Furthermore, the current study employs a different methodological approach than previous studies, which have tested relationships between constraints and motivation [e.g., (17)]. In contrast, we adopt a person-centered approach, which allows us to capture heterogeneity in the population by identifying distinct motivational profiles. This approach not only provides a more nuanced understanding of how different individuals perceive leisure constraints but also offers practical implications for designing targeted, segmented interventions (18). Although the person-centered approach has not been widely applied in leisure motivational research, studies in the general motivation literature [e.g., (18–21)] have demonstrated its promise. By applying this method, the aim was first to cluster individuals according to their motivation levels and then examine which constraints are perceived more intensively by each cluster. This analysis can reveal whether and how constraints interact with motivation, thereby further extending the hierarchical model of leisure constraints and the negotiation proposition. There has been no empirical research so far examining the individual motivation profiles in relation to the perception of leisure constraints.

### 1.1 Constraints to leisure participation

Crawford and Godbey (10) classified leisure constraints into three categories: intrapersonal, interpersonal, and structural. Intrapersonal constraints are internal ones; they include psychological traits and self-perceptions, such as lack of skills, perceived abilities, low body image, cultural and societal norms, and low fitness levels. These constraints influence mainly the preference for participation in a leisure activity, and as such, they are the most powerful in an individual's decision for leisure participation. They, in fact, block participation, rather than modify it (12). Interpersonal constraints are related to limited social interaction; behaviour is constrained by the inability to find partners for group participation or even conflicting social demands. Finally, the structural constraints are external ones, and they include factors such as limited time, financial resources, accessibility problems, and facilities-related issues. Crawford et al. (12) integrated these constraint types within a hierarchical decision-making framework. They proposed the “hierarchy of importance”, which ranks constraint types according to proximity to the individual decision-making. In their model, intrapersonal constraints are proposed as the most powerful, since they influence the preference for participation. They are the most likely to block participation. On the other hand, the structural constraints, which are the most distal, are more likely to modify rather than block participation. Finally, the interpersonal constraints can influence both the preference for participation and the actual behaviour. This framework is well theoretically established today. There is extensive published research that used the hierarchical model of leisure constraints to study behaviour in leisure, sport, recreation, and tourism settings [e.g., (5, 22–25)].

### 1.2 Motivation for leisure participation

The Self-Determination Theory (SDT), developed by Deci and Ryan (8, 26), provides a valuable and established framework to study and measure recreation motivation. SDT conceptualizes motivation along a continuum of self-determination, ranging from amotivation (a lack of intent or belief in one's competence) to various forms of extrinsic motivation, and ultimately to intrinsic motivation. Intrinsically motivated behavior is driven by personal interest, fun, enjoyment, and personal challenge (27–29); it usually leads to greater satisfaction and promotes optimal arousal and stimulation (30). Within the field of leisure studies, intrinsic motivation is often seen as more dominant than extrinsic motivation. This is largely because intrinsic motives lead to greater satisfaction, self-expression, and promote optimal arousal and stimulation (31).

On the other hand, extrinsic motivation leads to less self-determined behavior; individuals engage in activities not for fun and pleasure, but because they expect tangible benefits (e.g., improved appearance) and external rewards (9). A further categorization of extrinsic motivation defines it as introjected, identified, and integrated regulation. Extrinsic motivation, though perceived as externally regulated, can be internalized and can take more self-determined forms, such as identified regulation (engaging in an activity because it aligns with personal values) or integrated regulation (when external motivations are fully assimilated with the self). These motives can be related to the achievement of physical, psychological, and social benefits. In this case, individuals might not necessarily expect to experience excitement and pleasure when engaging in leisure activities, but they view participation as a worthwhile activity, since it is associated with health-related benefits (9). In this case, extrinsic motives might have the same positive behavioral and attitudinal outcomes as intrinsic motivation (e.g., increased satisfaction, engagement levels, commitment, etc.). In contrast, introjected regulation reflects less autonomous and more controlling forms of motivation (26). Introjected regulation represents the least autonomous motives, as individuals are driven primarily by tangible rewards, social recognition and acceptance, social norms, and even the avoidance of negative perceptions and feelings of guilt (32). Individuals engage in sport activities in compliance with “should” and “must” (33). Finally, in the amotivation stage, individuals have no reason for leisure participation; they are very likely to drop out (34). The literature on sport, leisure, and exercise motivation is extensive; it is not the objective of this paper to do a detailed review of this literature. A systematic review of the exercise motivation literature can be found in the studies of Standage and Ryan (35) and Manninen et al. (36).

### 1.3 The relationship between leisure constraints and motivation

Subsequent developments of the hierarchical model of leisure constraints included the “negotiation” and “balance” propositions (11). They were introduced to explain the reported non-significant or even positive relationships among dimensions of leisure constraints (mainly structural), and leisure participation [e.g., (37, 38)]. Two types of negotiation strategies were proposed by Jackson and Rucks (39): cognitive and behavioral. Cognitive strategies involve reframing constraints (e.g., rationalizing limited access), while behavioral strategies include tangible adjustments such as rearranging time commitments, developing necessary skills, altering social networks, or recalibrating leisure goals. These authors proposed that behavioral strategies were more prevalent and were chosen in response to specific types of constraints.

As previously noted, the outcome of this negotiation depends on the relative strength and interaction between perceived constraints and motivating factors (11). If motivation is strong, individuals might overcome constraints and reach participation. The concept of motivation has received some attention within the constraint's literature. Early studies by Carroll and Alexandris (37), Hubbard and Mannell (40), Stodolska (41), and Alexandris et al. (13) provided some evidence of the motivation's role within the hierarchical constraints model. Carroll and Alexandris (37) reported significant negative correlations between motivation and constraints. Hubbard and Mannell (40) reported that motivation appears to interact more strongly with negotiation strategies than directly with participation. Notably, they found that high motivation levels do not necessarily lead to a reduced perception of constraints, suggesting that some constraints—particularly intrapersonal ones—may precede and influence motivational states early in the decision-making process. On the other hand, Stodolska (41) offered a contrasting perspective through her qualitative study on immigrant leisure behavior, proposing that constraints may paradoxically function as motivators over time. Recent studies have applied the negotiation proposition to explain how constraints interact with perceived well-being (6), enjoyment (42), social capital (43), and self-efficacy (44). These factors can act as facilitators of the negotiation process, strengthening motivation to overcome constraints.

While the above studies examined motivation in relation to constraints, they did not use the Self-determination theory within the framework of the hierarchical model of leisure constraints in an integrated model. We propose that the position along this motivation continuum affects how individuals deal with constraints. Jackson et al. (11) proposed that the strength of this desire to participate, whether generated by a single motive or multiple factors, plays a crucial role in how individuals perceive or overcome constraints. In this line, more self-determined types of motivation, such as intrinsic or identified regulation, might be associated with greater persistence, higher satisfaction, and a stronger capacity to negotiate constraints. In contrast, individuals with low self-determination—those driven by external rewards or pressure—are more likely to perceive constraints as insurmountable, leading to reduced participation or complete disengagement.

### 1.4 Purpose of the study and research objectives

The current research uses a person-centered approach to examine motivations and constraints. Latent class analysis (LCA) was employed as the primary method, as it does not rely on the typical assumptions of traditional linear models. Additionally, LCA offers significant advantages, as it uses both a psychometric tool and a model-based clustering method, allowing for the identification of meaningful latent categories that can contribute to theoretical advancement. The resulting profiles represent new latent constructs formed by the interaction of multiple motivations, which can further support and refine the multiple motivation perspective by integrating both theoretical foundations and empirical evidence.

Based on the above discussion, the research objectives of this study were set as follows: (a) to cluster individuals according to their scores on the intrinsic and extrinsic motivation and amotivation; (b) to examine differences in the perception of constraints (intrapersonal, interpersonal, and structural) among the different motivation cluster groups.

## 2 Materials and research method

### 2.1 Procedure

The data were collected using an online questionnaire, which was posted on the research team's social media (Facebook, WhatsApp, Viber, and blogs), inviting people to complete the questionnaire. The goal was to draw a sample of the Greek adult general population, large enough to run the statistical analysis required in order to test the theoretical model. It has to be noted that our goal was not to have a representative sample of the general population of the country. Using the G*Power software, it was confirmed that the sample size exceeded the required minimum based on a 5% significance level, an 80% power level, and a medium effect size of.30 (45). With this process, we achieved a sample of three hundred and eighteen (*N* = 318) adult individuals. Informed consent was obtained from all participants before completing the questionnaire. Certain limitations associated with the sampling method must be acknowledged. The study utilized a convenience sampling method, meaning that participants were recruited based on accessibility rather than random selection. Consequently, the sample may not be representative of the study population, and the findings should be interpreted with caution. This sampling approach may introduce sampling bias and limit the generalizability of the results. Having a convenient sample is a common method in academic studies using samples of a general population, where the objective is to test theoretical models rather than provide representative results of the study population (e.g., the residents of a city) (4, 5). Once again, it should be emphasized that generalizations of the findings should be made with caution. The questionnaire included self-reported measures of recreational sport participation, constraints on participation, and motivation. Recreational sports were defined as sports and exercise activities performed during leisure time (4, 5). To ensure clarity, a list of seventeen sport activities—including walking for exercise—was provided, based on previous research [e.g., (4, 5)].

### 2.2 Participants

Concerning gender, the sample consisted of 49.5% men and 50.5% women. According to their educational level, the majority had completed secondary education (36.8%), while a substantial proportion reported holding a university degree (24.1%). Smaller, yet noteworthy segments included individuals with a master's degree (12.1%) and those who had attended a technical college (11.7%). In contrast, comparatively fewer participants had completed vocational training (7.9%) or primary education (7.3%). According to age, the largest proportion (24.2%) was individuals aged 56–65, followed closely by those aged 26–35 (22.3%) and 36–45 (21.7%) age groups. A smaller proportion is represented by individuals aged 18–25 (16.7%), while the age 46–55 and older than 56 age groups accounted for 7.9% and 7.2%, respectively.

### 2.3 Instruments

Motivation was measured with 12 items (46, https://selfdeterminationtheory.org/self-regulation-questionnaires) covering the Intrinsic Dimension (9 items), Extrinsic (3 items), and Amotivation (4 items). The instrument was translated into Greek by a professional translator, using a backtranslation technique (47). Seven-point Likert scales, ranging from “Totally disagree” to “Totally agree”, were used. The internal consistency reliability of the three subscales was found to be very good (*α* = .88, *α* = .90, *α* = .98, respectively).

Leisure constraints were assessed using the scale developed by Alexandris and Carroll (48), which has been successfully validated in Greek populations and extensively documented in the literature [e.g., (6, 15, 49)]. This scale includes sixteen items categorized into three dimensions: structural constraints (nine items), intrapersonal constraints (sixteen items), and interpersonal constraints (three items). Seven-point Likert scales, ranging from “Not important” to “Very important”, were used. The alpha scores of the three subscales were found to be very good (*α* = .91, *α* = .95, *α* = .91, respectively).

For each sub-dimension of the two questionnaires, mean scores of the relevant items were calculated to represent the composite score for each respondent. No cut-off criteria were applied; rather, the scores were interpreted on a continuum, with relative comparisons made between subdimensions.

### 2.4 Data analysis

Latent Class Analysis (LCA) was used as the statistical approach to uncover distinct groups or clusters, known as latent profiles or classes (50). These groups represent individuals who display similar response patterns. Participants categorized within the same latent class are considered to share comparable characteristics based on the parameters of the latent class model, which describes how they responded to specific items. The suitability of the model is evaluated using several indicators, such as the number of estimated parameters, the likelihood ratio statistic (L2), the Bayesian Information Criterion (BIC), the Akaike Information Criterion (AIC), degrees of freedom, classification accuracy, and bootstrapped *p*-values. Moreover, LCA allows for the inclusion of covariates, making it possible to examine how external factors relate to class membership (51). This method is especially advantageous because it is a flexible, multivariate analysis that does not depend on strict assumptions like normality or linearity.

**Figure 1:**
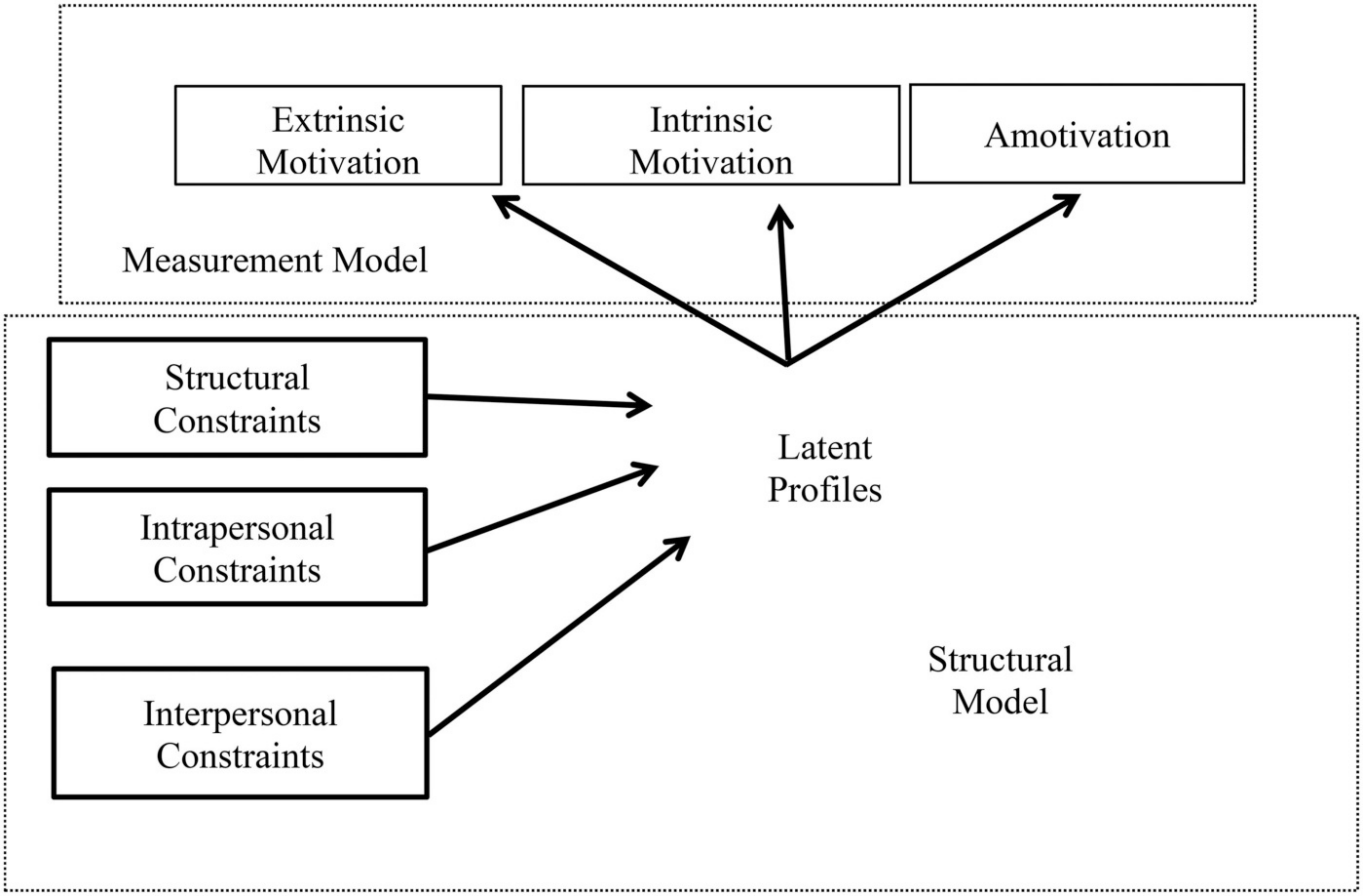
The structural and measurement model of latent class analysis. The Measurement model shows the way latent profiles are structured based on extrinsic motivation, intrinsic motivation, and amotivation and the structural model presents that structural constraints, intrapersonal constraints, and interpersonal constraints function as independent variables.

The analysis used a one-step method that simultaneously identifies latent classes and examines their associations with external covariates within the same model. This integrated approach helps reduce potential biases often found in conventional multi-step procedures. Figure 1 presents the full model that we evaluated, which is divided into two components: the measurement model and the structural model. The measurement model outlines how the profiles were represented, based on the motivations, whereas the structural model illustrates the covariation of the various constraints. The profiles that emerged based on extrinsic motivation, intrinsic motivation, and amotivation were evaluated to determine if they were predicted by structural, intrapersonal, and interpersonal constraints. Figure 1 represents the way the latent profiles are created. In selecting the optimal number of latent classes, we evaluated a balance of model simplicity, classification error rates, and the clarity of the class profiles. The final choice was guided not only by statistical criteria, including the number of estimated parameters and classification precision, but also by the practical significance and theoretical consistency of the identified classes.

## 3 Results

### 3.1 Latent class analysis (LCA)

**Figure 2:**
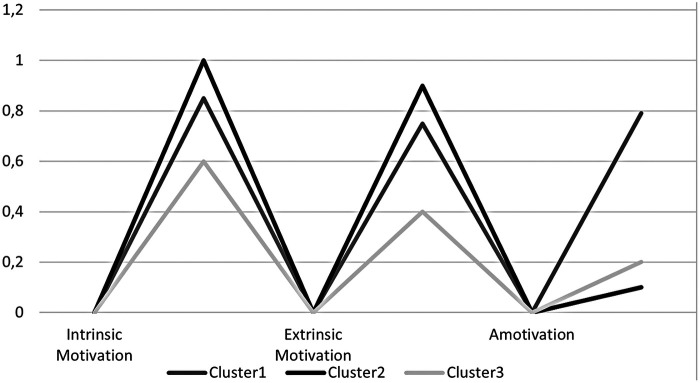
Conditional probabilities for the three clusters/profiles.

Table 1 presents the outcomes of the LCA analysis. The three-class solution was selected as the optimal model based not only on the BIC but also on minimal classification error, a parsimonious number of parameters, and strong interpretability of classes, and is acceptable. As the number of clusters increased, the model fit improved, but the gains diminished beyond the three-cluster solution. Although models with more clusters showed slightly better statistical fit, they also involved increased model complexity and higher classification error. Therefore, the three-cluster solution was deemed the most suitable model. Furthermore, the three-cluster model produced conceptually meaningful and distinct class profiles, supporting its selection as the best representation of the underlying population structure. These three clusters reflect distinct profiles, representing groups of individuals who share similar levels of extrinsic motivation, intrinsic motivation, and amotivation. Figure 2 displays the conditional probabilities, as they are linked to the three unique clusters or profiles, while Table 1 provides a descriptive summary of these profiles, highlighting their key characteristics and relative proportions.

**Table 1:** LCA results and model fit indices.

Models	Clusters	LL	BIC (LL)	Npar	Class.Err.
Model1	1-Cluster	−1909,17	4,192,873	65	0
Model2	2-Cluster	−1552,83	3,520,537	72	0.0001
Model3	3-Cluster	−1434,88	3,324,967	79	0.0066
Model4	4-Cluster	−1331,3	3,158,13	86	0.0069
Model5	5-Cluster	−1317,23	3,170,333	93	0.0069
Model6	0-Cluster				

The number of the models, different clusters, log-likelihood (LL), Bayesian Information Criterion (BIC), number of parameters (Npar), and classification error (Class. Err.) are reported.

Cluster 1/Profile 1 (38.65%) comprised people with medium intrinsic and extrinsic motivations and a high level of amotivation, and it is referred to as the “Medium Intrinsic-Extrinsic Motivations, High Amotivation” profile. Cluster 2/Profile 2 (33.39%) consisted of people with high intrinsic and extrinsic motivations and a low level of amotivation, and it is referred to as the “High Intrinsic-Extrinsic Motivations, Low Amotivation”. Cluster 3/Profile 3 (27.96%) included people with low levels of intrinsic and extrinsic motivation and medium amotivation, and it is referred to as the “Low Intrinsic -Extrinsic Motivations, Medium Amotivation” (Table 2). The three profiles were hypothesized to be predicted by constraints (independent variables). The following section examines this type of association.

**Table 2:** Qualitative description of the three clusters.

Profiles	Medium Intrinsic -Extrinsic Motivations, High Amotivation	High Intrinsic -Extrinsic Motivations, Low Amotivation	Low Intrinsic -Extrinsic Motivations, Medium Amotivation
Intrinsic motivation	Medium	High	Low
Extrinsic motivation	Medium	High	Low
Amotivation	High	Low	Medium

### 3.2 The three profiles as dependent variables

**Figure 3:**
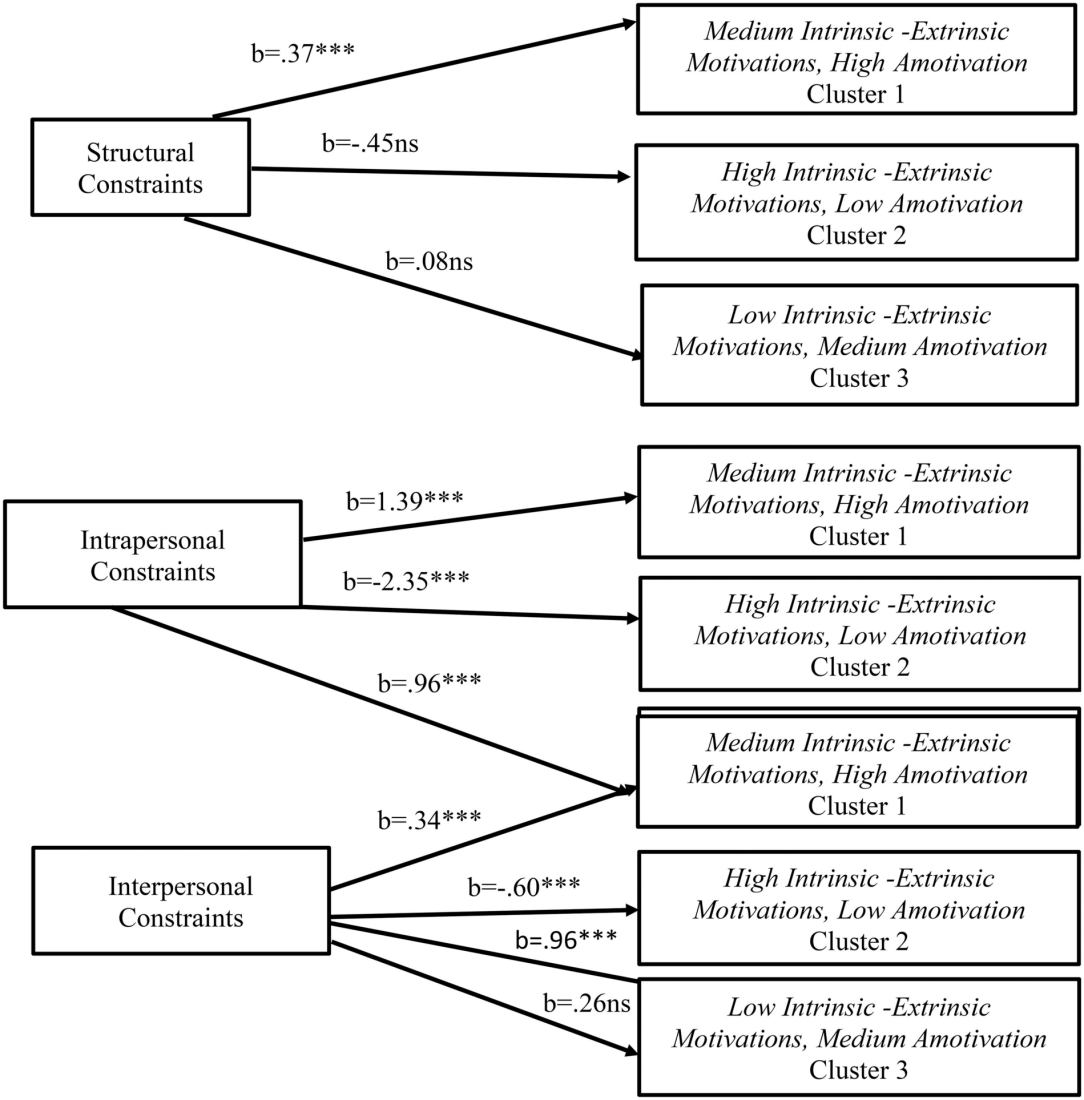
Constraints as independent variables for the three clusters. ***p < .001; ns, non-significant.

Furthermore, LCA demonstrated that the class memberships were predicted by constraints. Table 3 and Figure 3 display these effects. Structural constraints had a significant positive association (*b* = .37, *p* < .001), with the Medium Intrinsic -Extrinsic Motivations, High Amotivation profile (Cluster 1), but there was not any other association with High Intrinsic -Extrinsic Motivations, Low Amotivation (Cluster 2) (*b* = −.45, *p* > .05) and with Low Intrinsic -Extrinsic Motivations, Medium Amotivation (Cluster 3) (*b* = .08, *p* > .05). Further, the High Intrinsic -Extrinsic Motivations, Low Amotivation (Cluster 2) profile was negatively predicted by intrapersonal constraints (*b* = −2.35, *p* < .001) and by interpersonal constraints (*b* = −0.60, *p* < .001). Moreover, the Low Intrinsic -Extrinsic Motivations, Medium Amotivation (Cluster 3) was predicted positively by intrapersonal constraints (*b* = .96, *p* < .001), while there was no significant association with interpersonal constraints (*b* = .26, *p* > .05).

**Table 3:** The three latent classes as dependent variables of structural constraints, intrapersonal, and interpersonal constraints.

Constraints	Cluster 1	Cluster 2	Cluster 3
	Medium Intrinsic -Extrinsic Motivations, High Amotivation	High Intrinsic -Extrinsic Motivations, Low Amotivation	Low Intrinsic -Extrinsic Motivations, Medium Amotivation
Structural constraints	.37***	−0.45 ns	0.08 ns
Intrapersonal Constraints	1.39***	−2.35***	0.96***
Interpersonal Constraints	.34***	−0.60***	0.26 ns

ns = non-significant.

*** *p* < .001.

## 4 Discussion

This study aimed to cluster individuals according to their motivation scores and further examine these cluster groups' scores in the perception of leisure constraints. The results of the study first of all showed that the two dimensions of motivation and amotivation, as defined by the self-determination perspective, can be used as a basis for profiling recreational sport participants. Three distinct groups were revealed, which had statistically significantly different scores among them in terms of motivation.

In more detail, cluster 2 (33.39%) was the one with the highest motivation scores. It consisted of individuals with high intrinsic and extrinsic motivation scores and low amotivation. The high scores of both intrinsic and extrinsic motivations are a finding that can be interpreted based on the self-determination theory. As previously discussed, while intrinsic motivation plays the most important role in determining leisure behavior (31), extrinsic motivation, although perceived as externally regulated, can take more self-determined forms, such as identified regulation (engaging in an activity because it aligns with personal values) or integrated regulation (when external motivations are fully assimilated with the self). In this case, extrinsic motivation is internalized and guides behavior (52, 53). This is particularly applicable in the case of recreational sport participation, as previous research has also reported it (4, 49). As this cluster includes the most motivated individuals, we would expect that they would have the highest sport participation rates. In the next level, our LCA analysis indicated that this cluster had the strongest negative associations with intrapersonal constraints. As previously noted, according to the hierarchical model of leisure constraints, the intrapersonal are the most powerful constraints are the most likely to block rather than modify participation (11), Subsequently, this strong and significant negative association shows that individuals who are highly motivated (intrinsically and extrinsically) are more like to overcome intrapersonal constraints and get more engaged with recreational sport participation. Once again, it should be noted that intrapersonal constraints include mainly psychological constraints and self-perceptions related to low self-esteem, negative body image, social norms, and negative beliefs/attitudes towards sport participation. Some of these constraints might relate to negative past experiences.

Cluster 3 (27.96%) included mainly individuals with low levels of intrinsic and extrinsic motivation. This is the cluster with the least motivated participants. Our analysis indicated that Cluster 3 was positively predicted by intrapersonal constraints, while there was no significant relationship with interpersonal constraints. Once again, these results are in line with the hierarchical model of leisure constraints and the balance proposition. They indicate that low-motivated individuals are mainly influenced by intrapersonal constraints. In this case, the intensity of leisure constraints perception is stronger than motivation. Due to the low motivation levels, individuals are not able to successfully negotiate and overcome leisure constraints. Subsequently, the interaction brings constraints as “winners” against motivation. It is worth noting that in this cluster, neither interpersonal nor structural constraints have significant associations.

Finally, Cluster 1 (38.65%) comprised individuals with medium intrinsic and extrinsic motivation and a high level of amotivation. We would expect that higher scores in amotivation would be associated with low scores in motivation (especially the intrinsic), as there has been evidence in the study of Alexandris et al. (13). This group was obviously the least distinctive and homogeneous one. It might include individuals who still participate in recreational sport activities, although not being highly motivated (since they report high scores in the amotivation dimension); but they still understand the physical and psychological benefits of participation, which motivate them to a specific level. This cluster might also include individuals who used to participate, but they have dropped out, due to their low motivation levels (they cannot “convince” themselves to do it anymore). In this line, the moderate scores in the two motivation dimensions might not be strong enough to drive individuals to keep on participating or to start participating. On a different interpretation, these results can also reflect the limitations of the self-reported measures of sport behavior. In any case, this is a group that needs to be further studied. Our analysis also showed that the three dimensions of leisure constraints did not have negative associations with motivation within this cluster, which is also an unexpected finding. The negotiation proposition (11) can be applied here to interpret the results. These individuals were probably capable of negotiating their constraints, although they had reported them, due to their moderate levels of intrinsic and extrinsic motivation and despite the high scores in the amotivation dimension. However, this is a finding that also deserves more research. It would be interesting to further test the recreation participation behavioral profile of this group to draw further conclusions.

These results can be discussed with reference to policy implications. First, they propose that research on motivation and constraints can help researchers and practitioners to understand recreational sport participation behavior. Segmenting individuals according to their motivation levels and perception of constraints can guide policy and marketing strategy. Individuals with low levels of intrinsic motivation are the most likely to be influenced by the perception of intrapersonal constraints and either do not start participating or drop out. Intrinsic motivation is about free choices, experiencing fun, excitement, and positive social interaction during sport participation. It is therefore important for sport development officers and community leaders to provide exercise environments that foster and satisfy the above needs. The choice of the appropriate sport activities is one of the key points. The delivered recreation programs should fit with community interests, considering issues such as their members' age, sport experience, culture, and preferences. Providing the right sport settings is important for addressing intrapersonal constraints, which were the most influential for decreasing motivation. Exercise environments that promote social support during exercise can help individuals to successfully negotiate their psychological constraints. Research has shown that providing group programs is not enough, however (6). Social support can be the key point. Research has shown that exercise settings that promote the development of social capital are the most motivated ones (54). The development of trust among the exercise members, the mutual exchange of help and support, and the development of social networks will help individuals to be more motivated and overcome intrapersonal and interpersonal constraints. Since our study also showed that extrinsic motivation can play a role in overcoming leisure constraints, we can also propose promoting the physical and psychological benefits of sport participation; furthermore, role modeling and positive body images can be used to motivate behavior, although they can originally be considered as less self-determined motives. They can be internalized if participation is associated with positive experience and the achievement of personal goals. Finally, the role of social support, including formal and informal social networks, can help towards overcoming leisure constraints, as a recent study showed (5).

### 4.1 Study limitations and suggestions for future research

First, the study used a cross-sectional design. This means that results do not reflect causal relationships among variables. A longitudinal methodological approach is required to study stability and change of behavior over time and determine with confidence the causal relationships among variables.

As previously noted, this study used a convenient Greek sample, with a size that it was adequate to test the theoretical model used. It is expected that a convenience sample has a large sampling error, and subsequently, the results cannot be generalized to the study population. The results are indicative and not representative. Future studies should test the model in different cultural settings. A cross-cultural study, including populations from other countries, could examine the role of personal and societal values, which might influence the perception of intrapersonal and interpersonal constraints.

In a final note, it should be noted that while the present study examined the interaction between constraints and motivation, it did not include any behavioral aspects of the participants, such as frequency or intensity of participation, or any attitudinal aspects, such as involvement and loyalty levels. It will be interesting for future studies to incorporate behavioral or attitudinal variables as well, in order to test an integrated decision-making model, as proposed by the hierarchical model of leisure constraints (55).

## 5 Conclusion

In conclusion, the current study is one of the very few that examine motivation profiles of individuals, in relation to the perception of leisure constraints, using the theoretical frameworks of the self-determination perspective and the hierarchical model of leisure constraints. The person-centered approach that we adopted allowed us to identify three distinct motivational profiles, based on the two motivation and the amotivation dimensions, providing partial support for the self-determination theory. This methodological approach gave us the chance to study the interactions between motivation and constraints within the motivation segments. These interactions were proposed and discussed in previous studies, but there have not been empirical attempts to test them within specific motivation clusters. The results supported the important role of intrinsic but also extrinsic motivation in successful negotiating and overcoming intrapersonal constraints, which are the most powerful ones in determining engagement in recreational sport participation. The role of extrinsic motivation should be emphasized since, in the context of recreational sports, it is equally important as intrinsic motivation through the individual internalization processes.

## Data Availability

The raw data supporting the conclusions of this article will be made available by the authors, without undue reservation.
